# Use of term reference infants in assessing the developmental outcome of extremely preterm infants: lessons learned in a multicenter study

**DOI:** 10.1038/s41372-023-01729-x

**Published:** 2023-08-04

**Authors:** Charles E. Green, Jon E. Tyson, Roy J. Heyne, Susan R. Hintz, Betty R. Vohr, Carla M. Bann, Abhik Das, Edward F. Bell, Sana Boral Debsareea, Emily Stephens, Marie G. Gantz, Carolyn M. Petrie Huitema, Karen J. Johnson, Kristi L. Watterberg, Ricardo Mosquera, Myriam Peralta-Carcelen, Deanne E. Wilson-Costello, Tarah T. Colaizy, Nathalie L. Maitre, Stephanie L. Merhar, Ira Adams-Chapman, Janell Fuller, Michelle E. Hartley-McAndrew, William F. Malcolm, Sarah Winter, Andrea F. Duncan, Gary J. Myer, Stephen D. Kicklighter, Myra H. Wyckoff, Sara B. DeMauro, Anna Maria Hibbs, Barbara J. Stoll, Waldemar A. Carlo, Krisa P. Van Meurs, Matthew A. Rysavy, Ravi M. Patel, Pablo J. Sánchez, Abbot R. Laptook, C. Michael Cotten, Carl T. D’Angio, Michele C. Walsh, Eunice Kennedy Shriver National Institute of Child Health and Human Development Neonatal Research Network, Richard A. Polin, Martin Keszler, Angelita M. Hensman, Elisa Vieira, Lucille St. Pierre, Robert T. Burke, Barbara Alksninis, Andrea Knoll, Mary L. Keszler, Teresa M. Leach, Elisabeth C. McGowan, Victoria E. Watson, Nancy S. Newman, Bonnie S. Siner, Elizabeth Roth, Angelia Williams, Brenda B. Poindexter, Kurt Schibler, K. Tanya E. Cahill, Cathy Grisby, Kristin Kirker, Sara Stacey, Sandra Wuertz, Ronald N. Goldberg, Ricki F. Goldstein, Patricia L. Ashley, Deesha Mago-Shah, Joanne Finkle, Kimberley A. Fisher, Kathryn E. Gustafson, Caitlin Stone, Matthew M. Laughon, Janice Bernhardt, Janice Wereszczak, Jennifer Talbert, Alexandra Bentley, Laura Edwards, Ginger Rhodes-Ryan, Donna White, David P. Carlton, Yvonne Loggins, Diane Bottcher, Sheena L. Carter, Salathiel Kendrick-Allwood, Maureen Mulligan LaRossa, Judith Laursen, Colleen Mackie, Amy Sanders, Gloria Smikle, Lynn Wineski, Andrew A. Bremer, Rosemary D. Higgins, Stephanie Wilson Archer, Amir M. Khan, Kathleen A. Kennedy, Andrea F. Duncan, Elizabeth Allain, Julie Arldt-McAlister, Fatima Boricha, Allison G. Dempsey, Carmen Garcia, Donna J. Hall, Janice John, M. Layne Lillie, Karen Martin, Georgia E. McDavid, Shannon L. McKee, Michelle Poe, Kimberly Rennie, Tina Reddy, Shawna Rodgers, Daniel K. Sperry, Sharon L. Wright, Leif D. Nelin, Jonathan L. Slaughter, Sudarshan R. Jadcherla, Christopher Timan, Patricia Luzader, Melanie Stein, Rox Ann Sullivan, Helen Carey, Stephanie Burkhardt, Mary Ann Nelin, Erna Clark, Kristi Small, Jacqueline McCool, Lindsay Pietruszewski, Jessica Purnell, Kyrstin Warnimont, Laura Marzec, Bethany Miller, Demi R. Beckford, Hallie Baugher, Katelyn Levengood, Nancy Batterson, Jill Tonneman, Krystal Hay, Brittany DeSantis, Dennis Wallace, Jeanette O’ Donnell Auman, Margaret Crawford, Jenna Gabrio, Jamie E. Newman, Lindsay Parlberg, Kristin M. Zaterka-Baxter, David K. Stevenson, M. Bethany Ball, Valerie Chock, Dona Bahmani, Barbara Bentley, Maria Elena DeAnda, Anne M. DeBattista, Beth Earhart, Lynne C. Huffman, Casey E. Krueger, Ryan E. Lucash, Heather Taylor, Hali E. Weiss, Namasivayam Ambalavanan, Kirstin J. Bailey, Fred J. Biasini, Stephanie A. Chopko, Monica V. Collins, Shirley S. Cosby, Kristy A. Domnanovich, Chantel J. Jno-Finn, Morissa Ladinsky, Mary Beth Moses, Tara E. McNair, Vivien A. Phillips, Julie Preskitt, Richard V. Rector, Kimberlly Stringer, Sally Whitley, Sheree York Chapman, Heidi M. Harmon, Karen J. Johnson, Mendi L. Schmelzel, Jacky R. Walker, Claire A. Goeke, Sarah E. Faruqui, Diane L. Eastman, Michelle L. Baack, Laurie A. Hogden, Megan M. Henning, Chelsey Elenkiwich, Megan Broadbent, Sarah Van Muyden, Robin K. Ohls, Conra Backstrom Lacy, Sandra Sundquist Beauman, Mary Ruffner Hanson, Jean R. Lowe, Elizabeth Kuan, Eric C. Eichenwald, Barbara Schmidt, Haresh Kirpalani, Soraya Abbasi, Aasma S. Chaudhary, Toni Mancini, Jonathan Snyder, Kristina Ziolkowski, Ronnie Guillet, Satyan Lakshminrusimha, Anne Marie Reynolds, Rosemary L. Jensen, Joan Merzbach, William Zorn, Osman Farooq, Gary J. Myers, Mary Rowan, Diane Prinzing, Melissa Bowman, Ann Marie Scorsone, Kyle Binion, Constance Orme, Premini Sabaratnam, Alison Kent, Rachel Jones, Elizabeth Boylin, Emily Li, Jennifer Kachelmeyer, Kimberly G. McKee, Kelly R. Coleman, Brenna Cavanaugh, Luc P. Brion, Diana M. Vasil, Sally S. Adams, Frances Eubanks, E. Rebecca McDougald, Lara Pavageau, Pollieanna Sepulveda, Alicia Guzman, Elizabeth Heyne, Lizette E. Lee, Azucena Vera, Jillian Waterbury, Cathy Twell Boatman, Bradley A. Yoder, Mariana Baserga, Roger G. Faix, Stephen D. Minton, Mark J. Sheffield, Carrie A. Rau, Shawna Baker, Susan Christensen, Sean D. Cunningham, Jennifer O. Elmont, Becky Hall, Erika R. Jensen, Manndi C. Loertscher, Trisha Marchant, Kandace M. McGrath, Hena G. Mickelsen, Galina Morshedzadeh, D. Melody Parry, Kelly Stout, Ashley L. Stuart, Kimberlee Weaver-Lewis

**Affiliations:** 1 Department of Pediatrics, McGovern Medical School at The University of Texas Health Science Center at Houston, Houston, TX USA; 2 Department of Pediatrics, University of Texas Southwestern Medical Center, Dallas, TX USA; 3 Department of Pediatrics, Division of Neonatal and Developmental Medicine, Stanford University School of Medicine and Lucile Packard Children’s Hospital, Palo Alto, CA USA; 4 Department of Pediatrics, Women & Infants Hospital, Brown University, Providence, RI USA; 5 Social, Statistical and Environmental Sciences Unit, RTI International, Research Triangle Park, Greensboro, NC USA; 6 Social, Statistical and Environmental Sciences Unit, RTI International, Rockville, MD USA; 7 Department of Pediatrics, University of Iowa, Iowa City, IA USA; 8 Center for Clinical Research and Evidence-Based Medicine, University of Texas Houston McGovern Medical School, Houston, TX USA; 9 University of New Mexico Health Sciences Center, Albuquerque, NM USA; 10 Children’s Memorial Hermann Hospital, Houston, TX USA; 11 Division of Neonatology, University of Alabama at Birmingham, Birmingham, AL USA; 12 Department of Pediatrics, Rainbow Babies & Children’s Hospital, Case Western Reserve University, Cleveland, OH USA; 13 Department of Pediatrics, Nationwide Children’s Hospital, The Ohio State University College of Medicine, Columbus, OH USA; 14 Department of Pediatrics, University of Cincinnati College of Medicine, Cincinnati, OH USA; 15 Emory University School of Medicine, Department of Pediatrics, Children’s Healthcare of Atlanta, Atlanta, GA USA; 16 Department of Pediatrics, University of New Mexico, Albuquerque, NM USA; 17 Department of Pediatrics, University of Buffalo, Buffalo, NY USA; 18 Department of Pediatrics, Duke University, Durham, NC USA; 19 Department of Pediatrics, Division of Neonatology, University of Utah School of Medicine, Salt Lake City, UT USA; 20 Department of Pediatrics, Pennsylvania Hospital, Philadelphia, PA USA; 21 University of Rochester School of Medicine and Dentistry, Rochester, NY USA; 22 Department of Pediatrics, Wake Medical Center, Raleigh, NC USA; 23 Department of Pediatrics, University of Pennsylvania, Philadelphia, PA USA; 24 Eunice Kennedy Shriver National Institute of Child Health and Human Development, National Institutes of Health, Bethesda, MD USA; 25 Division of Neonatology, College of Physicians and Surgeons, Columbia University, New York, NY USA; 26 Alpert Medical School of Brown University and Women & Infants Hospital of Rhode Island, Providence, RI USA; 27 Case Western Reserve University, Rainbow Babies & Children’s Hospital, Cleveland, OH USA; 28 Cincinnati Children’s Hospital Medical Center, University Hospital, and Good Samaritan Hospital, Cincinnati, OH USA; 29 Duke University School of Medicine, University Hospital, University of North Carolina, Duke Regional Hospital, and WakeMed Health & Hospitals, Durham, NC USA; 30 Emory University, Children’s Healthcare of Atlanta, Grady Memorial Hospital, and Emory University Hospital Midtown, Atlanta, GA USA; 31 Eunice Kennedy Shriver National Institute of Child Health and Human Development, Bethesda, MD USA; 32 McGovern Medical School at The University of Texas Health Science Center at Houston, Children’s Memorial Hermann Hospital, and Memorial Hermann Southwest Hospital, Houston, TX USA; 33 Nationwide Children’s Hospital, The Abigail Wexner Research Institute at Nationwide Children’s Hospital, Center for Perinatal Research, The Ohio State University College of Medicine, The Ohio State University Wexner Medical Center, and Riverside Methodist Hospital, Columbus, OH USA; 34 RTI International, Rockville, MD USA; 35 Stanford University, El Camino Hospital, and Lucile Packard Children’s Hospital, Stanford, CA USA; 36 University of Alabama at Birmingham Health System and Children’s Hospital of Alabama, Tuscaloosa, AL USA; 37 University of Iowa and Sanford Health, Iowa City, IA USA; 38 University of Pennsylvania, Hospital of the University of Pennsylvania, Pennsylvania Hospital, Children’s Hospital of Philadelphia, and Virtua Voorhees Hospital, Philadelphia, PA USA; 39 University of Rochester Medical Center, Golisano Children’s Hospital, and the University of Buffalo Women’s and Children’s Hospital of Buffalo, Rochester, NY USA; 40 University of Texas Southwestern Medical Center, Parkland Health & Hospital System, and Children’s Medical Center Dallas, Dallas, TX USA; 41 University of Utah Medical Center, Intermountain Medical Center, McKay-Dee Hospital, Utah Valley Hospital, and Primary Children’s Medical Center, Salt Lake City, UT USA

**Keywords:** Paediatrics, Neurological manifestations

## Abstract

**Objective:**

Extremely preterm (EP) impairment rates are likely underestimated using the Bayley III norm-based thresholds scores and may be better assessed relative to concurrent healthy term reference (TR) infants born in the same hospital.

**Study design:**

Blinded, certified examiners in the Neonatal Research Network (NRN) evaluated EP survivors and a sample of healthy TR infants recruited near the 2-year assessment age.

**Results:**

We assessed 1452 EP infants and 183 TR infants. TR-based thresholds showed higher overall EP impairment than Bayley norm-based thresholds (O.R. = 1.86; [95% CI 1.56–2.23], especially for severe impairment (36% vs. 24%; *p* ≤ 0.001).

Difficulty recruiting TR patients at 2 years extended the study by 14 months and affected their demographics.

**Conclusion:**

Impairment rates among EP infants appear to be substantially underestimated from Bayley III norms. These rates may be best assessed by comparison with healthy term infants followed with minimal attrition from birth in the same centers.

**ClinicalTrials.gov ID:**

Term Reference (under the Generic Database Study): NCT00063063

## Introduction

Follow-up assessments of extremely preterm (EP) infants are difficult to perform and interpret for multiple reasons. As for other assessments [1], the expectations or biases of unblinded examiners may have an important effect on the findings. This problem can be minimized by including a concurrently assessed reference group of term infants and assuring that the examiners are masked to gestational age, perinatal complications, and findings of any prior follow-up assessments [2–5].

Another issue is the appropriate comparison group of term infants. One approach is to compare EP infants to term infants matched for maternal age, ethnicity, income, education, marital status, insurance status, etc. This approach has been used in efforts to identify the independent effects of perinatal factors on outcomes. However, matching is logistically difficult, quite likely to be incomplete, and precludes assessment of how adverse socioeconomic factors and their interactions with biological or medical factors compromise the outcomes of EP infants. A better understanding of all these factors is needed to develop improved methods to reduce rates of impairment among EP born children. For these reasons, a comparison to healthy term infants may be preferred in deciding which EP infants should be considered to have a developmental impairment based on the child’s capabilities irrespective of the extent to which these impairments result from medical, socioeconomic, or other factors [6].

Additional issues include the choice of the developmental test and whether its norms are fully appropriate in designating which EP infants should be considered impaired [2, 7–11]. While the Bayley Scales of Infant and Toddler Development (Bayley III) have been widely used, multiple investigators have reported that the impairment rates are likely to be underestimated in applying its norms [2, 7–14]. Moreover, it is difficult to assure that the examiners in all centers perform the Bayley III assessments in the same way that the assessments were performed when the Bayley III was normed.

For all these reasons, the NICHD Neonatal Research Network (NRN) undertook the study described below to assess EP and a concurrent sample of healthy term reference (TR) infants examined by the same blinded and certified examiners in the same centers at two years corrected age. We hypothesized that the proportion of EP infants with developmental impairment based on standard deviations (SDs) from the mean for the TR sample would be higher than that based on Bayley III norms. If so, we hoped to identify threshold values for Bayley III scores based on our term reference infants that would be more appropriate than those based on the Bayley III norms for categorizing EP infants as impaired in NRN centers.

## Methods

The study was conducted in 15 NRN centers between January 2017 and March 2020. The addition of the TR infants to the follow-up assessments was approved by each center’s Institutional Review Board (IRB). Consent was obtained in accordance with each study site’s IRB requirements.

### Design

To augment the reliability of the assessments and reduce the likelihood that examiner expectations would affect the scores, the TR and EP infants in the study were assessed concurrently by examiners not informed of their gestational age at birth or their prior clinical or developmental findings.

### Eligibility and sampling

The eligible EP infants were inborn at NICHD NRN centers and <27 weeks gestation by best obstetric estimate. Infants with at least one Bayley III composite score at the 24-month follow-up visit were included in the analysis.

Eligible TR infants met the following criteria assessed using the medical record: singleton birth at 39 0/7-40 6/7 weeks gestation by best obstetric estimate; birth weight appropriate for gestational age; no resuscitation at birth; absence of congenital anomalies or other abnormalities on physical examination; benign neonatal course with all care given in a low risk nursery and no neonatal problem delaying discharge home; and parent(s) willing and able to come into the clinic. Exclusion criteria included major central nervous system disorder (e.g., cerebral palsy, deafness, blindness or the effects of major insults identified by parent report or medical records [e.g. meningitis or traumatic brain injury before two years]), child protective services custody, parental incarceration, and parental psychosis.

Our goal was to assess one healthy term infant for every fifth EP survivor at 22–26 months corrected for prematurity in the same center to evaluate 180 total TR infants in a one-year study. (See Sample Size and Power.) Recruitment of each healthy TR infant began shortly (e.g. 1–2 months) before the corresponding EP infant’s scheduled assessment. If the EP infant was lost to follow-up, the TR infant was still to be assessed.

Center coordinators used medical records to identify and attempt to recruit the first healthy term infant born on or after the expected due date of the index EP infant. The potential value of developmental testing was emphasized in recruiting. The methods of contact (letter, text, phone call) and incentives used to promote participation (e.g. up to $100 plus parking or $50 plus cab fare) varied as allowed by the individual site’s IRB. When a parent or guardian declined participation or missed two scheduled clinic visits, the coordinator contacted the next eligible infant’s parent or guardian by delivery time and date until one agreed for her child to participate within the testing window.

To assess the representativeness of the TR sample with all term births in the NRN centers we requested the information for all term infants born in NRN hospitals during the study period. To further characterize the sample of TR infants we qualitatively contrasted the estimates on available data from the Bayley-III normative data.

### Measurement and comparisons

Certified Bayley III examiners, trained to reliability and re-evaluated annually, provided assessments at each NRN center [15, 16]. The Bayley III was administered to Spanish-speaking children by either a Spanish-speaking evaluator or an English-speaking evaluator with a translator. Means and SD’s of Bayley III scores among the TR infants were used to determine new thresholds for each of the Bayley III composites (cognitive, language, and motor) to indicate three levels of impairment: (1) Normal/mild, a score greater than or equal to 1 SD below the mean; (2) Moderate, a score between one and two SD below the mean; and (3) Severe, a score lower than 2 SD below the mean. Application of these new cut points to the EP infants determined the proportion falling into each category [15, 16].

### Statistical analysis

Generalized linear multilevel models compared the proportion of infants in each category using the norm-based vs. TR thresholds, accounting for clustering of infants within centers. Levels of impairment were analyzed using an ordinal logistic model and dichotomous variables (moderate/severe vs. normal/mild) were analyzed using a binomial model. Analyses were conducted using SAS version 9.3.

### Sample size and power

Assuming a 5% rate of impairment based on Bayley III manual norms, a 15% rate of impairment based on thresholds derived from the reference group [2], and an intraclass correlation of 0.05 due to center membership, a sample of *N* = 180 provided 91% power to detect a 10% absolute difference in impairments > 2 S.D.’s below the mean with alpha = 0.05. Given prior, annual rates of enrollment for EP infants we anticipated that recruiting EP to TR in a 1:5 ratio would result in *N* = 180 within one year.

## Results

**Fig. 1:**
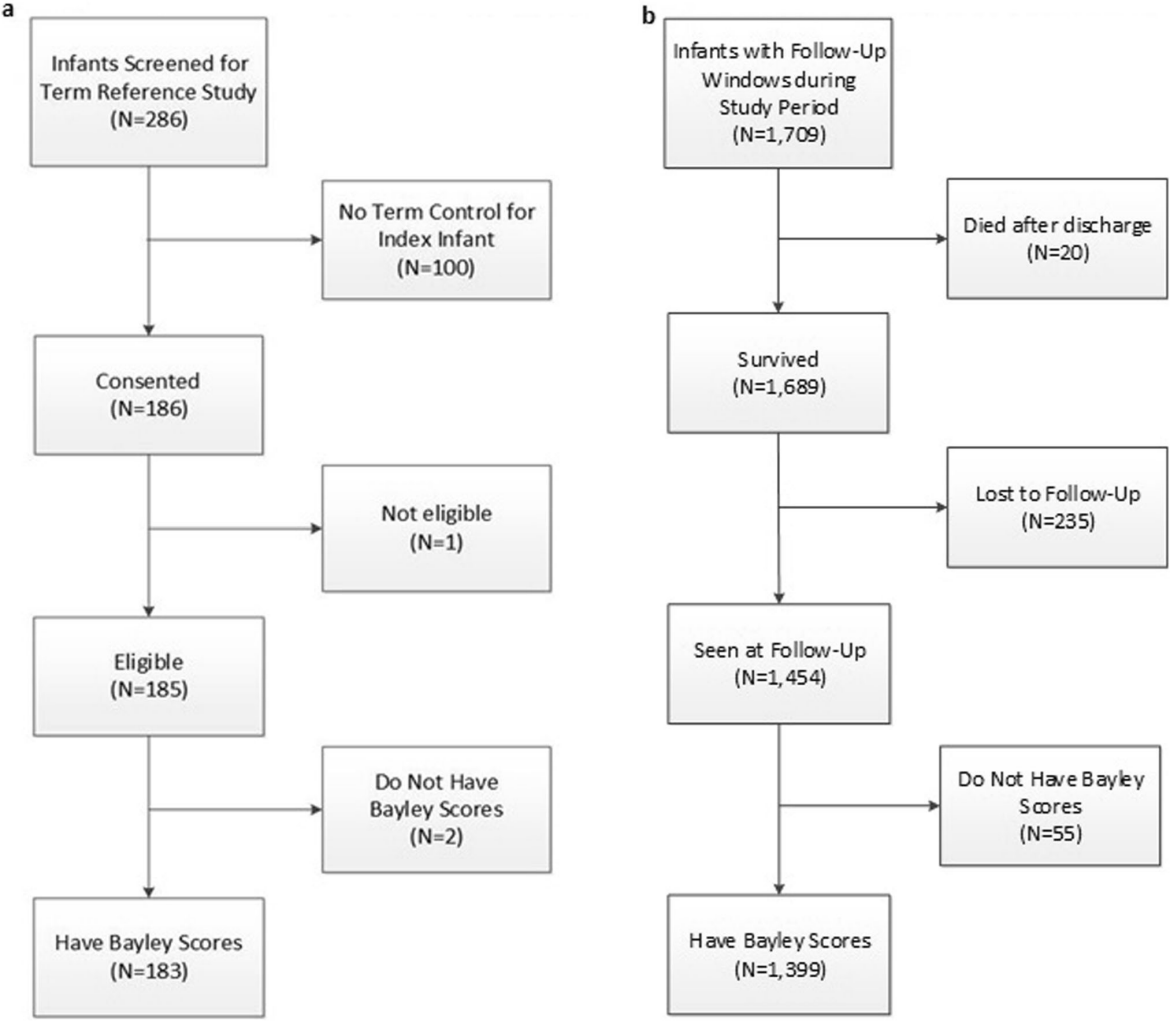
Sampling diagram. Sample selection for term-reference (**a**) and pre-term (**b**) infants.

A total of 1452 EP infants (86% of survivors at 2 years) were evaluated during the time required to accrue and successfully assess 183 TR infants (Fig. 1). This accrual of TR infants took longer than expected (38 versus 24 months with an accrual ratio of 1:8 versus 1:5. Based on querying site coordinators, reasons for slower accrual than expected varied among centers but included difficulty accessing the medical records in some hospitals that were not owned by the university, problems contacting the parents using letters (as required by some IRBs), variable incentives for participation allowed by the IRBs, parental inconvenience, transportation problems, and in one center, contract negotiations between the university and an affiliated hospital.

### Demographic comparison of the TR sample and EP infants

Mothers of TR infants were more often White, married and more highly educated. Mothers of EP infants were more often African-American (Table 1).

**Table 1:** Sociodemographic and medical characteristics of term and preterm infants.

Characteristic	Term (*N* = 183)	Preterm (*N* = 1452)
	*N* (%)	*N* (%)
**Maternal**		
Maternal age…mean (SD)	30.13 (5.9)	28.72 (6.1)
Race		
African American	51 (28)	580 (40)
White	115 (63)	742 (51)
American Indian/Alaskan Native	0 (0)	11 (1)
Asian, Native Hawaiian, or Other Pacific Islander	5 (3)	44 (3)
More than One Race	4 (2)	30 (2)
Unknown	8 (4)	45 (3)
Ethnicity		
Hispanic or Latino	37 (20)	239 (16)
Not Hispanic or Latino	145 (79)	1199 (83)
Unknown	1 (1)	14 (1)
Gravidity…mean (SD)	2.56 (1.6)	2.96 (2.2)
Parity…mean (SD)	1.91 (1.1)	2.20 (1.4)
Marital status		
Married	108 (59)	647 (45)
Not married	74 (40)	801 (55)
Unknown	1 (1)	4 (0)
Education		
8^th^ grade or less	2 (1)	41 (3)
9^th^ to 12^th^ grade	8 (4)	105 (7)
High school diploma	31 (17)	274 (19)
Trade or technical school	6 (3)	165 (11)
Partial college/associate degree	39 (21)	387 (27)
College degree	41 (22)	193 (13)
Graduate degree	53 (29)	93 (6)
Unknown	3 (2)	194 (13)
**Neonatal**		
Gestational age…mean (SD)	39.25 (0.6)	24.88 (1.1)
Birth weight…mean (SD)	3406.8 (359.8)	753.2 (165.1)
Sex		
Male	92 (50)	711 (49)
Female	91 (50)	741 (51)
**Follow-Up**		
GMFCS^a^ level		
Normal/Level 0	167 (91)	961 (66)
Possible Level 1	0 (0)	16 (1)
Level 1	14 (8)	330 (23)
Level 2	0 (0)	77 (5)
Level 3	0 (0)	23 (2)
Level 4	0 (0)	16 (1)
Level 5	0 (0)	23 (2)
Unknown	2 (1)	6 (0)
Moderate/severe CP^a^		
Yes	0 (0)	110 (8)
No	181 (99)	1341 (92)
Unknown	2 (1)	1 (0)
Vision impairment (Bilateral blind with no/some functional vision)		
Yes	0 (0)	16 (1)
No	182 (99)	1436 (99)
Unknown	1 (1)	0 (0)
Hearing impairment (Any impairment with or without amplification)		
Yes	1 (1)	39 (3)
No	181 (99)	1375 (95)
Unknown	1 (1)	38 (3)

^a^*GMFCS* Gross Motor Function Classification System.

*CP* Cerebral Palsy.

### Demographic comparison of the TR sample, term births at NRN hospitals and the Bayley III normative population

The information that NRN hospitals provided about their term births was incomplete and varied between hospitals, resulting in uncertainty in how the TR sample differed from all children born at term in these centers. Since the TR group included only healthy infants, modest differences would be expected. However, in the 11 centers where the information was provided (Table 2), there were 30% fewer TR children with Medicaid/public insurance and 24% more with private insurance.

**Table 2:** Demographic characteristics for all term births at participating sites^a^.

Variable (Number of centers)	Survey (*N* = 101,422)	Study (*N* = 170)
	*n* (%)	*n* (%)
**Birth Weight (13)**^**b**^	***n*** = **100,277**	***n*** = **155**
<1500 g	14 (0)	0 (0)
1501-2500 g	3005 (3)	0 (0)
2501-4000 g	89872 (90)	149 (95)
> 4000 g	7305 (7)	6 (4)
**Sex (14)**	***n*** = **101,422**	***n*** = **170**
Male	50631 (50)	84 (49)
Female	50778 (50)	86 (51)
Ambiguous	18 (0)	0 (0)
**Race**^**c**^ **(11)**	***n*** = **80,922**	***n*** = **137**
White	39761 (49)	78 (57)
African American	28241 (35)	44 (32)
American Indian/Alaskan Native	620 (1)	0 (0)
Asian	5051 (6)	5 (4)
Native Hawaiian or other Pacific Islander	316 (0)	0 (0)
More than one race	920 (1)	3 (2)
Unknown	5955 (7)	7 (5)
**Ethnicity**^**c**^ **(14)**	***n*** = **101,422**	***n*** = **170**
Hispanic	26785 (26)	39 (23)
Non-Hispanic	66961 (66)	130 (76)
Unknown	7676 (8)	1 (1)
**Insurance**^**d**^ **(11)**	***n*** = **77,166**	***n*** = **106**
Medicaid/public insurance	46358 (60)	32 (30)
Self-pay/uninsured	1969 (3)	9 (8)
Private	27461 (36)	64 (60)
Unknown	443 (1)	0 (0)
Other	735 (1)	1 (1)

^a^All analyses exclude one due to lack of data on demographics by gestational age.

^b^Birthweight analyses also exclude two due to missing data.

^c^Race analyses also exclude five centers due to missing data.

^d^Insurance analyses also exclude eight centers due to missing data.

The data for our TR sample were compared with the data provided for the normative Bayley III sample at age two years gathered by the test company (i.e. *n* = 100 children at 24 months). The TR sample differed from the Bayley normative sample with respect to percent who were Hispanic (20 vs 16%), African-American (28% vs 14%) and parents with ≥ 16 years of education (51% vs. 29%). Surprisingly, the Bayley III Technical Manual did not characterize the normative sample in terms of marital or insurance status, did not report the proportion of children approached for inclusion who did not participate, or indicate any measures to blind the evaluators to any unfavorable social, medical, or biologic factors that might influence scores [17].

### Bayley III scores for TR and EP infants

The mean composite cognitive, motor, and language scores were 83.9, 83.3, and 80.2, respectively, for the EP infants and 97.5, 98.2, and 97.9, respectively for the TR group (Table 3). As expected, with the deliberate inclusion of children with developmental problems in the Bayley normative sample, the SDs were less for our healthy TR sample for the Cognitive Composite (11.2, 95% CI 10.2–12.5) and the Motor Composite (10.9, 95% CI 9.9–12.2) than for the Bayley normative sample (SD = 15 for all composites). The SD for the Language Composite in the TR sample was 16.0 (95% CI 14.5–17.9), similar to the manual-based SD (15). The composite score SDs for the EP infants ranged from 15.1–17.4.

**Table 3:** Bayley III scores among term reference (TR) and extremely preterm (EP) infants.

	Term Infants			Pre-Term Infants		
	*N*	Mean	SD	*N*	Mean	SD
Cognitive Composite Score	182	97.5	11.2	1446	83.9	15.1
Language Composite Score	180	97.9	16.0	1409	80.2	17.4
Expressive Language Scaled Score	180	9.5	3.0	1391	6.7	3.0
Receptive Language Scaled Score	180	9.8	3.0	1405	6.6	3.1
Motor Composite Score	178	98.2	10.9	1403	83.3	16.2
Fine Motor Scaled Score	180	10.3	2.0	1412	7.9	3.0
Gross Motor Scaled Score	179	9.1	2.2	1385	6.7	2.7

The ranges for all three Bayley composite scores based on norm-based thresholds were ≥ 85, 70–84 and 55–69 respectively for all three Bayley III composite scores. Using term-reference data resulted in ranges for normal/mild, moderate and severe thresholds of ≥86.21, 75–86.20 and 63.73–74.97 for the Cognitive Composite, ≥87.31, 76.38–87.30 and 65.45–76.37 for the Motor Composite, and ≥81.91, 65.88–81.90 and 49.85–65.87 for the Language Composite. The Bayley III score thresholds for severe impairment ( < 2 SDs below the mean) for Cognitive and Motor Composites were thus were 5-6 points higher than for the Bayley normative sample. However, the Language Composite threshold was approximately 3 points lower.

### Comparison of impairment rates

Term-reference-based impairment thresholds resulted in higher overall rates of moderate/severe impairment (i.e. impairment on *any* one of the Cognitive, Motor or Language Composites Scores) (Table 4 bottom). The same was true for impairment identified using just the Cognitive and Motor Composites. Given the larger, term-reference-estimated SD for the Language Composite, the norm-based thresholds resulted in higher rates of moderate/severe language impairment (Table 4). As evident in Table 4, the differences between the Manual and Term Reference based rates of moderate and severe impairment were largely to the difference in severe impairment. A second set of post-hoc analyses adjusting for maternal education, language spoken at home and age at assessment did not substantially alter these results.

**Table 4:** Proportion of EP infants designated moderately/severely impaired using norm-based versus term reference based threshold scores for impairment.

Variable	Manual norm-based impairment threshold	Term reference based impairment threshold	Term reference vs. norm-based	
	*N* (%)	*N* (%)	OR (95% CI)^a^	*p*-value
**Moderate/Severe Impairment**				
Cognitive				
Normal/Mild	825 (57)	642 (44)	2.01 (1.69,2.39)	<0.001
Moderate/Severe	621 (43)	804 (56)		
Language				
Normal/Mild	625 (44)	716 (51)	0.68 (0.57,0.82)	<0.001
Moderate/Severe	784 (56)	693 (49)		
Motor				
Normal/Mild	776 (55)	662 (47)	1.60 (1.34,1.92)	<0.001
Moderate/Severe	627 (45)	741 (53)		
**Level of Impairment (3 categories)**				
Cognitive				
Normal/mild	825 (57)	642 (44)	2.50 (2.10,2.96)	<0.001
Moderate	395 (27)	478 (33)		
Severe	226 (16)	326 (23)		
Language Composite				
Normal/mild	625 (44)	716 (51)	0.60 (0.50,0.71)	<0.001
Moderate	412 (29)	379 (27)		
Severe	372 (26)	314 (22)		
Motor Composite				
Normal/mild	776 (55)	662 (47)	2.48 (2.08,2.96)	<0.001
Moderate	373 (27)	321 (23)		
Severe	254 (18)	420 (30)		
**Overall Developmental Impairment (i.e. Cognitive, Motor or Langauage Composite)**				
Moderate/severe impairment				
Normal/mild	603 (43)	457 (32)	1.86 (1.56,2.23)	<0.001
Moderate/severe/	806 (57)	965 (68)		
Level of impairment				
Normal/mild	603 (44)	457 (33)	2.72 (2.29,3.22)	<0.001
Moderate/	456 (33)	436 (31)		
Severe	326 (24)	497 (36)		

^a^Odds ratios are based on models accounting for clustering of participants by site. Higher odds ratios indicate greater impairment when using the term reference cut points and lower odds ratios indicate less impairment when using the term reference cut points.

## Discussion

We assessed Bayley III scores at two years adjusted age for EP infants and TR infants born in the same NRN centers and examined by the same assessors who had been trained to reliability [18] and were blinded to gestational age at birth, perinatal events, and prior follow-up findings. The mean composite cognitive, motor, and language scores were 83.9, 83.3, and 80.2, respectively, for the EP infants and 97.5, 98.2, and 97.9, respectively, for the TR group.

The mean Bayley III composite scores for our TR group were lower than for term control infants in some other studies [2, 3, 12] despite the high proportion of well-educated TR mothers. This finding may be due to greater socioeconomic disadvantages; our sample contained a higher proportion of Hispanic, African American, and Medicaid-insured children than the term controls in most other studies.

More EP infants had moderate or severe cognitive and motor impairments (composite scores more than 1 or 2 SDs below the mean, respectively) using the scores for TR sample (SD = 10.9–11.3) than the Bayley III normative sample (SD = 15.0). These differences are likely due in part to the different referent populations assessed. To avoid under-identification of impaired EP children, children with major congenital anomalies, perinatal problems, or postnatal insults likely to affect development [2, 3, 6] were systematically excluded from our TR sample. A different approach was used for the Bayley III normative sample, in which 10% of the children had such problems as Down’s syndrome, cerebral palsy and language impairments [17]. While a reference population that includes the full spectrum of child development is desirable for some purposes [6], this approach would likely understate the proportion of impaired preterm infants when threshold scores 1 or 2 SDs below the mean for the Bayley III normative population are used to designate impairments. Accordingly, Sharp and DeMauro [7], among others, suggest that different and higher threshold Bayley III scores are needed.

As hypothesized, the overall proportion of EP infants with a cognitive, motor, or language impairment based on a composite score at least 1 SD below the mean for our TR group was higher than that based on Bayley III normative population (68 vs. 57%, *p* < 0.01)). The difference was particularly marked for severe impairment (one or more composite scores at least 2 SDs below the mean; 36 vs. 24%, *p* ≤ 0.001). An unexpected finding was that the proportion of EP infants with composite language scores lower than 1 SD below the mean based on our TR sample was not higher than for the Bayley normative sample. This finding reflects a relatively high SD (16.0) for the TR language scores which may well be due to a high proportion of Hispanics and marked heterogeneity in parental education among the TR parents and a greater influence of education on language than on cognition or motor scores.

### Study limitations

The approach in most follow-up studies to assessing EP infants and designating their impairment rates involves some uncertainty about the reliability and inadvertent bias of the examiners as well as the appropriateness of the Bayley normative sample. While our study facilitated blinded Bayley III assessment of EP and TR infants by the same carefully trained and certified examiners, our sample of healthy TR infants was not sufficiently representative of healthy term infants in NRN centers to establish clear impairment thresholds for outcomes in the NRN. Our findings for insurance coverage and parental education indicate that attempts to recruit such infants two years or more after birth are difficult and prone to selection bias. Caregivers who had concerns about their child’s development may have been more likely to participate, a problem that would cause us to underestimate the degree to which impairment rates were underestimated using Bayley norms. Future efforts to recruit a representative sample of healthy term infants may be more successful if these infants are enrolled in the neonatal period with special measures to maintain rapport with the parents and achieve high follow-up rates through the age of assessment [19].

The need to minimize bias in assessing EP infants may be achieved more simply by including a convenience sample of term reference controls and blinding the evaluators to gestational age, medical history, and any prior follow-up assessments. However, it is unclear whether the Bayley IV Scales address the need emphasized by Sharp and DeMauro [7] among others to establish higher impairment thresholds for the Bayley III Scales. While the Bayley-IV has superceded the Bayley-III the current results are still informative. The Bayley-IV Technical Manual states, “Because most of the Bayley-4 is a revision of the previous edition, most of the validity evidence reported in the research related to the Bayley-III is still relevant….”. (p. 37) [20].

Accurate identification and monitoring of impairment rates in EP infants is critical for multiple reasons, including provision of appropriate services and parental counselling for individual infants, planning their long term education and rehabilitation, testing perinatal interventions in proper clinical trials, and evaluating care and outcomes within and across different perinatal centers over time. The impairment rates identified among EP and other high-risk infants have been almost always assessed by examiners well aware of the infants’ risk factors and prior assessments. Yet,the need for blinded assessors and concurrently assessed control patients should not be assumed to be less important to assure unbiased assessments in follow-up clinics than in other settings. High priority should be given in neonatal follow-up programs to developing effective methods to meet this need and to define appropriate impairment thresholds for EP infants.

## Data Availability

Data collected at participating sites of the NICHD Neonatal Research Network were transmitted to RTI International, the data coordinating center (DCC) for the network, which stored, managed and analyzed the data included in this study. On behalf of the NRN, RTI International had full access to all the data in the study and take responsibility for the integrity of the data and accuracy of the data analysis. The content is solely the responsibility of the authors and does not necessarily represent the official views of the National Institutes of Health. Data reported in this paper may be requested through a data use agreement. Further details are available at https://neonatal.rti.org/index.cfm?fuseaction=DataRequest.Home.
